# A Conservation Ethic and the Collecting of Animals by Institutions of Natural Heritage in the Twenty-First Century: Case Study of the Australian Museum

**DOI:** 10.3390/ani1010176

**Published:** 2011-02-15

**Authors:** Timothy Ikin

**Affiliations:** Department of Museum Studies, School of Letters, Arts and Media, Faculty of Arts, University of Sydney, Sydney, NSW 2006, Australia; E-Mail: tiki3446@uni.sydney.edu.au

**Keywords:** collecting, animal conservation, biological collections, Australian museum

## Abstract

**Simple Summary:**

It is a core task of collecting institutions like museums to take examples of animals and preserve them as specimens in collections. In the twenty-first century, museums are equally the places where research is conducted and education is promoted in the service of conservation of animals in an era of the decline of biodiversity. In this paper, the balance of co-operation between collecting of animals by museums and the promotion and scientific pursuit of conservation of fauna in those museums is considered. As a “challenge” to museum science, it is considered in the context of Australia's oldest museum, and its policy and practice in the current century.

**Abstract:**

Collecting of animals from their habitats for preservation by museums and related bodies is a core operation of such institutions. Conservation of biodiversity in the current era is a priority in the scientific agendas of museums of natural heritage in Australia and the world. Intuitively, to take animals *from* the wild, while engaged in scientific or other practices that are supposed to promote their ongoing survival, may appear be incompatible. The Australian Museum presents an interesting ground to consider zoological collecting by museums in the twenty-first century. Anderson and Reeves in 1994 argued that a milieu existed that undervalued native species, and that the role of natural history museums, up to as late as the mid-twentieth century, was only to make a record the faunal diversity of Australia, which would inevitably be extinct. Despite the latter, conservation of Australia's faunal diversity is a key aspect of research programmes in Australia's institutions of natural heritage in the current era. This paper analyses collecting of animals, a core task for institutions of natural heritage, and how this interacts with a professed “conservation ethic” in a twenty-first century Australian setting.

## 1. Introduction

Interest in collecting, especially in the natural sciences, has recently arisen in the history and philosophy of science and other fields. Robert Kohler has substantially published on the history of biology, especially field science and collecting in America [1]. He recently argued that histories of collecting have been more about collections rather than collecting *per se*; that is, being about the objects, and not the means to amass them [2]. The reason for the latter is based on, Kohler asserts, that past scholarship has focused on meanings and semiotics of objects as against the means by which the objects were actually collected [2]. Kohler further argues that an “implicit bias” against collecting in science, that it is in fact historically “pre-science”, or preparation for scientific research [2]. Nonetheless, collecting and preservation of fauna has been a continued practice for the natural history museums of the world, including Australia.

The collecting of animals for preservation is a familiar description of one of the functions of a modern museum. Museum collecting of animals is a multi-modal practice and is currently conducted with a number of objects in mind: taxonomic collecting (or permanent collection-building); research or problem-based collecting; collecting for exhibition and public programs. The latter aims are pursued via a number of means: field collecting (e.g., by museum scientists or curators), receipt of donations (e.g., from bequest by an individual), exchange (e.g., with other Museums in Australia or the World), and receipt of research collections (e.g., from collaboration with scientific institutions like the Australian agency the C.S.I.R.O.).

Despite what era that a scientist, collector or institution may belong to, the target of such acquisition for any collector of fauna is the “specimen”, an individual representative of a species retained by the collector often in multiples. Anne Larsen presents specimens as “manageable pieces of the natural world” for the eighteenth and nineteenth century naturalist, that were however, not “natural” but constructed by naturalists to answer their needs [3]. The specimen remains the final object of collecting by museums and other institutions of natural heritage in the twenty-first century. The acquisition and retaining of a preserved specimen of an animal by institutions that are publicly identified with efforts to conserve Australia's fauna in their own habitat may appear initially contradictory [4].

This paper explores how zoological collecting (and the policy governing such) by museums in Australia interacts with the museum policy and practice aiding conservation of biodiversity and environmental planning. The Australian Museum will be the focus of my examination, as it is the oldest museum in Australia, which has an array of accessible sources which allow for examination of animal collecting. The Australian Museum was founded *ca.* 1827, and found a permanent home at its current site in the mid 1850s. It presently houses collections of over an estimated 16 million specimens and objects [5]. Similar to Phillip Rainbow's discussion of the *Discovery* marine biology collections (made from 1900–1904) [6], zoological collections like that of the Australian Museum are important in the history of zoology and the natural sciences. Rainbow identifies the *Discovery* collections as spanning a time of “transition” during which the perceived use of such collections has changed [6]. Zoological collections like those of Australia that were founded in the early period of European colonization are similar to the latter as they span the eras of “encyclopaedic” collecting through to the research-driven concerns of our time. This research is part of a larger study in pursuit of a doctoral thesis about the history and contemporary role of zoological collecting at the Australian Museum, being completed by the current author at the University of Sydney.

The Australian Museum portrays its contemporary collecting in the following manner on its website:

Over the last two centuries the methods of, and reasons for, collecting in museums have changed significantly. Museum science and research is no longer the Victorian model of encyclopaedic collections and ‘cabinets of curiosities’ [7].

If this is the case, then what characterizes contemporary collecting in the museum context, and what consequences does that have for their use as tools in conservation science?

Museums as the agents of specimen collecting are equally familiar as places which promote awareness of biodiversity and conservation of species in their own environment, in the current era. As noted by Anne Larsen Hollerbach regarding the early nineteenth century [8] and Peter Davis regarding the twentieth century [9], zoological collecting, whether directly in the field by museum scientists or via exchange, requires the capture, removal and death and preservation of that animal at some stage of the collecting process. On the surface, ongoing collecting by museums of natural heritage in our era could quite possibly be in opposition to a commitment to conservation of faunal diversity.

A recent review of the commitment to conservation by collecting institutions in 2004, identified museums more with their “dioramas” and exhibits as a means of educating the public about conservation rather than recognizing the potential utility of specimens as conservation tools [10]. A substantial literature exists that museum collections are useful and often crucial in detecting decline in populations of threatened species, and other trends in biodiversity. Winston Ponder, of the Australian Museum, describes faunal collections as “huge databases” that mostly have “accumulated over a long period, so they provide an historical perspective impossible to obtain with contemporary field surveys” [11]. Further, The Australian Government's *National Collaborative Research Infrastructure Strategy* (cited in the *Australian Museum Science Research Strategy*, discussed further below) considers biological collections “key research infrastructure” [12].

## 2. Australian Fauna & Museums

Collecting as a tool of the biological scientist in fact came under criticism in the late 19th century as laboratory disciplines rose to represent “top practice” in academic departments and government funded institutions, and the study of zoology moved from museums to universities. According to Colin Finney, it was by as early as the 1890s that biology dominated natural science in Australian universities, not natural history [13], which is historically allied with collecting of specimens and not the laboratory. Collecting by museums in Australia did not halt, however, but continued and have built the vast collections available to scientists at the Australian Museum and elsewhere. Anderson and Reeves argue that collecting by museums and other institutions in Australia was affected by a milieu that “consistently undervalued native flora and fauna” [14] and that the purpose of museum collecting was to preserve a record of Australian species, but not to promote their conservation in the wild, a view prevalent until the mid-twentieth century [14]. We see similar views represented by a key museum official in Australia. J.W. Evans, director of the Australian Museum 1954–1966, wrote in 1963 in the journal *Museum* on the “functions of natural history museums”:

The pace of the destruction of forest and scrub by fire, bulldozer and plough is accelerating. Consequently, an urgent problem faces museums in the south Pacific (though of course it is not one peculiar to them) is to endeavour to make as comprehensive collections as possible from threatened areas while there is yet time. Otherwise, many animals, particularly those confined to restricted environments may become extinct without leaving any trace of their former existence [15].

We see just less than 30 years later that the concern of the Australian Museum with the loss of species has a different but substantial implication. For example in the annual *Corporate Report* year ending 1991: “Before we can determine the impact that humans are having on other species that share this dry continent with us, we need to understand the natural history of Australia and our neighbouring countries. And we need to know species still survive, and where, before we can talk sensibly about loss of biodiversity in this region of the Globe” [16]. The latter treatment of loss of Australian fauna differs from Evans' perspective in that museum activities are part of the effort to preserve biodiversity rather than as “record makers” of the fauna that has vanished or soon will. Within the museum model, collecting of specimens are the means to gain knowledge of native fauna and to gauge what species are threatened, rather than to make collections in the hope of cataloguing Australia's fauna as they become extinct. Linden Gillbank in 1988 observed the change in direction over the decades of the twentieth century in Australia and Australasia: “Imperially inspired herbarium and museum collections have become integral components of national and international conservation-oriented biological projects” [17]. As presented by the Australian Museum itself on its website, quoted above, the reasons for collecting have changed substantially since the foundation of the Museum.

If zoological collecting is now parts of crucial work in biodiversity conservation, certain pressures are placed on how that collecting is conducted. It is the need for a record of animal species, and their populations and distribution, past and present that is needed to be able to determine decline or change in native fauna, which is what is termed in this paper, the “*challenge of conservation*” for a collecting institution in the twenty-first century. Within the museum model, collecting of specimens for preservation is a core function of such institutions, but conservation of biodiversity entails seeking the continuation of species in the wild—can museum collecting of animals and a conservation ethic co-exist simultaneously?

## 3. Zoological Collecting Now

Collecting of fauna by museums, with its associations with “old fashioned” natural history approaches, may have contributed to its perceived “counterintuitive” role in conservation programs in contemporary museums. Museum collections in Australia may have the further unwarranted association of being dusty, old and established decades or centuries ago, with no contributions to be made now; the “museum as mausoleum” stereotype [18]. They are in fact constantly growing resources, and have been continually added to by collectors throughout the 20th century and now. For example, the Herpetology collection at the Australian Museum had the majority of its growth (80%) from collecting in the 1970s and after [19]. Robert Kohler argues that the era currently is that of “project collecting” where specimens are gathered to solve a problem, and the days of vast expeditions or intensive survey are now gone [1]. Even collection-building activities conducted by the Australian Museum are tailored to address specific gaps in zoological collections, rather than large scale amassing of specimens. We see that attitude reflected in the *Collection Development Strategy* for natural sciences collections: “In recent years Collection Managers have conducted collecting trips with the aim of developing the collections to complement acquisitions received from donations and research. These collections have targeted geographic areas or habitats that have been poorly collected, common species that are poorly represented in the collections and taxa that are required for current research projects by taxonomic experts at the AM and other institutions” [19].

From the earliest annual reports of the Australian Museum, collecting for the year was reported, firstly listing acquisitions of the museum as a whole and then later for each department. The later 1990s generally saw a reduction of such reporting to a single number to represent acquisitions for the entire museum. While collecting is ongoing in contemporary operations at the Australian Museum (acquisitions in 2007–2008 were almost double that of 2006–2007 with >400,000 acquisitions [20,21]), its place in the public reporting of the institution has reduced. It is quite rare that collecting *per se* is identified as of central concern in the functioning of the museum. In the *Report* for 1986–1987 it is stated that “Acquisition and preservation of collections are central to the achievement of the mission” [22], but similar statements are quite rare otherwise. Throughout the 1990s “maintenance and improvement” of collections is a far more common phrase in Annual Reports, and is broader than collecting specifically.

While it may not be presented in a plain “numerical” manner, animal collecting is still presented in the annual reports of the Museum, but are more often placed in context with the scientific projects supporting biodiversity and conservation research. Collecting is perhaps less concerned with the sheer amassing of specimens but more with the reason that collecting was conducted or use it will be put to in the future. In the 2007–2008 *Annual Report*, samples collected on 12-day field trip to Tathra area on the New South Wales South coast “will provide additional information on the biota and diversity of Australian marine environments” [20]. Given that museums in Australia have been collecting animals in an ongoing fashion as part of its function, throughout the last century and now, we must look to how such collecting is reconciled with a twentieth and twenty-first century animal conservation ethic which is essentially concerned with the continuation of species in their own environments.

## 4. Conservation in the Australian Museum

The 21st century visitor to an institution such as the Australian Museum would be quite sure of the role that the Museum plays in biological research, conservation and ecology. The Museum has and does present itself as an agency which influences debate on environmental issues, and via its vast collections is in a prominent place to do so in Australian society. It is specific about its position today in the *Science Research Strategy*: “Museum research is directly relevant to several of the NSW Government's state-wide targets for natural resource management under the State Plan, in particular, those relating to biodiversity and water” [23]. The Museum further identifies with the *NSW Biodiversity Strategy* (1999) [24] as a plan concerned with loss of flora and fauna.

Scientific interest and concern for issues the environment and conservation emerged formally with the establishment of the Department Environmental Studies in 1968. The Departments of Vertebrate and Invertebrate Terrestrial Ecology grew out of Environmental Studies in 1978. Currently, departments of the Museum are split into research divisions for marine and terrestrial biota, and natural science collections with a department for Arachnology, Entomology, Herpetology, Icthyology, Malacology, Mammology, Marine Invertebrates, Mineralogy, Ornithology and Paeleontology [25]. Conservation research is carried across many of the departments and collections. The Australian Museum certainly engages in biodiversity and environmental surveys that do not involve collecting exclusively, as they have for the past 20 years. For example, current radio tracking and banding of the White Ibis, which is moving out of its natural habitat in the Murray Darling Basin and into more urban areas [26].

### The Challenge of Conservation

As noted by Lyn Barber, in the era post-Darwin, Zoologists could (and can) no longer conceive of and collect the entirety of the animal world [27], as biodiversity is in flux. The latter is one of the contemporary arguments for continued collecting in academic correspondence. Kevin Winker likens faunal collections to “functional biological libraries” but asks the worth of a library that stops acquiring any books [28]. Henri Oullet, past Chief of Vertebrate Zoology at the National Museum of Natural Sciences in Ottawa, argues that while the collecting of specimens appears contradictory to conservation concerns, it is often only from specimens that we can gain the answers to conservation problems [4].

To be able to detect a decline in a species, there must be a prior historical record of the population and distribution of that species to enable biologists to observe any change. It is usually impossible to reconstruct such a record without a physical collection [29]. In the twenty-first century biological collecting must be representative if it is to be of utility to biologists. The Australian Museum, in its recent *Natural Sciences Collection Development Strategy 2007–2012*, requires that collecting be mindful of an array of representative criteria: geographic representation, temporal representation, taxonomic representation, specimen representation [19]. Collecting must, therefore, be ongoing over time if it is to be comprehensive and useful in conservation planning and research. Biological collections must have *temporal representation*. In addressing temporal representation the *Strategy* identifies that a:

collection with good temporal representation allows users to map the spatial movements and presence of animals over time. Temporal data is important for tracing the introduction of invasive species, the decline of threatened and endangered species and monitoring environmental change. This requires a time series of identifiable specimens in the collection [19].

We see similar issues addressed elsewhere, as in the Australian Museum *Science Research Strategy*, where it is assumed to be fundamental to the Museum's research is the “Building of collections through space and time” [23], similar concepts are also included in the recent 2008–2013 *Corporate Strategic Plan* [30].

## 5. The Australian Museum: Responses to the Challenge of Conservation

The Australian Museum has put forward a number of other policy statements and strategies where they bring together collecting and conservation concerns. On their own website they identify the overall usefulness of biological collections:

Museum collections reduce the need for scientists to collect new specimens or objects when conducting research. This is especially important when research is focused on endangered or vulnerable species. The collections are of increasing importance in a changing world where our natural environments are being rapidly degraded [31].

By creating and building on animal collections museums provide a centralized record of fauna, and does not require further impact on biological communities from collecting by individual scientists or groups. But to achieve such a useful centralized record, museums must collect animal specimens in an ongoing manner. The Australian Museum advises that: “Collecting must be undertaken in such a manner and involving such numbers, as to have no deleterious effects on the survival of local or regional biota or communities, nor have negative impacts on other societies or cultures” [32].

Later in the *Collection Management Policy* they identify that coverage of the collections must be improved by exchange with other institutions, or by receipt of collections from research projects:

In order to minimize the impact of collecting activities on existing biotic communities, every effort will be made to increase the quantity, quality and geographic coverage of our collections by exchange with other scientific institutions, or by acquiring collections resulting from research or monitoring programs in other organizations [32].

The deposit of *voucher specimens* following the completion of a research survey or other project is regularly identified in the related literature as an important part of any biodiversity survey. As defined by J.T. Huber they “are the supporting evidence for the occurrence of a species at a particular time and place. When doubt exists as to the identity of a species, voucher specimens permit re-examination to check or correct previous identifications” [33]. Pat Hutchings (2007) from the Australian Museum notes that specimens collected in a survey of Port of Sydney for marine pests can be used as baseline for future studies, and such specimens are also available to taxonomists for revision of marine taxa that can, in turn, inform future surveys of potential new pest species [34]. The receipt of voucher specimens from surveys of Australian and other fauna are a means for museums to grow their zoological collections from collecting activities that directly serve conservation research and planning. As suggested in the Australian Museum's *Natural Science Collection Development Strategy*, specimens received from Museum biological research projects may provide spatial or other representation that is outside the scope of the Museum's traditional taxonomic collecting [19].

The Museum directly prioritizes acquisition of those specimens which are needed to “solve urgent problems” in conservation of animals and other issues: “Priority will be given to acquisitions which are required to solve urgent problems, such as issues relating to the environment, conservation of animals, systematics or to contribute to an improved understanding of critical cultural issues and consistent with the Australian Museum Science Research Strategy” [32].

The policy and strategy recommendations of the Australian Museum divide into two categories: those that seek to reduce the impact of ongoing collecting on existing biota, and those that specifically employ collecting to address conservation needs. To provide appropriate representation in time and space, and to be useful as a benchmark for future research and conservation planning, ongoing collection must occur. Relying only on receipts from specific research projects, or only private and institutional donations and exchanges will most likely not provide the representative coverage required. The challenge of conservation for Australian museums is to balance collecting conducted in both modes.

## 6. Conclusions

Conservation has been a concern of The Australian Museum and other institutions in Australian since the second half of the twentieth century. Zoological collecting is perceived differently in the current era than in the era of foundation of Australia's museums, as a proposed means of preventing loss of biodiversity rather than merely recording it. It provides the historical record of faunal biodiversity via ongoing collecting, an endeavor which must occur virtually indefinitely as biodiversity is constantly changing under ecological and human-made pressures. Also, they collect animal specimens to address specific or urgent issues of conservation in the immediate environment. Such collecting occurs over a short-term time scale, but also further builds collections within the taxa of concern. Collecting of animals by museums in the current era needs to be wary of the tension between the need for ongoing collecting to maintain representativeness (and thus, utility to biological science) and the need to engage such collecting in a manner that does not harm the fauna which are of concern. It is an ongoing challenge for the Australian Museum in its policy and practice to mediate such a tension in its ongoing role as a collecting institution in Australia.

Zoological collecting has continued as a practice in the conduct of biological science within the museum model, despite pressures such as those from changing perspectives as to what represented modern biology in the late nineteenth and early twentieth century. Due to such continuation of collecting, there exist historical records of faunal diversity that allow for comparison with contemporary surveys, the specimens from which can also be added to collections in a co-operative relationship. Collecting and conservation may appear to be contradictory, given that zoological collecting in its traditional sense involves the acquisition and lodging of a preserved rather than live specimen in a collection, out of its habitat. In the museum model, however, collecting of animals provides the permanent record for biologists of the future to examine the state of Australian biodiversity.
